# Anatomical investigations on root, stem, and leaf of *Gentiana olivieri* Griseb

**DOI:** 10.4103/0973-1296.75877

**Published:** 2011

**Authors:** Canan Yağci Tüzün, Mehmet Cihat Toker, Gülnur Toker

**Affiliations:** *Ankara University, Science Faculty, Department of Biology, Tandogan, 06100, Ankara, Turkey*; 1 *Gazi University, Faculty of Pharmacy, Department of Pharmacognosy, 06330 Etiler, Ankara, Turkey*

**Keywords:** *Gentiana olivieri*, Gentianaceae, root, stem and leaf anatomy

## Abstract

**Background:**

*Gentiana olivieri* Griseb. (Afat) (Gentianaceae), which has many bioactive compounds is used as antidiabetic, hepatoprotective, digestive aid, antidepressant, and antianemic in traditional medicine.

**Materials and Methods:**

Root, stem, and leaf sections of *G. olivieri* were taken free hand or by sliding microtome and examined on light microscope.

**Results:**

Anatomical characters of the species were observed to be similar to the usual features of Gentianaceae anatomy.

**Conclusion:**

Intraxylary phloem, which was primarily the distinguishing feature between Gentianoideae and Menyanthoideae sub-families was observed in *G. olivieri* roots.

## INTRODUCTION

*Gentiana olivieri* Griseb. (Gentianaceae) spreads out from the middle East (Turkey, Iran, Iraq, Afghanistan) to East Asia (to Tian Shan) and this plant is an Irano-Turanien element. This perennial herbaceous plant grows on limestone, marl, or clay slopes and grassy meadows at an altitude of 350–2300 m. Height of this plant is 10–30 cm from a basal rosette. Root stock is sheated with a fibrous collar at apex. Dark blue-purple flowers bloom on April–July. Seeds are brown, ellipsoid, and 0.8–1 mm long. Testa is thin reticulate.[1]

Some species of the genus *Gentiana* are well known for centuries mainly in the Far East countries. Various herbal preparations such as Longdan and Qinjiao contain these plants and are used to cure hepatitis, constipation, rheumatism, pain, hypertension, anorexia and inflammation.[2,3]

*G. olivieri* has also been used for centuries as a medicinal plant in traditional folk medicines because of its secoiridoid, flavonoid, and alkaloid contents. This plant is known as “Afat” in Turkey, “Agher” and “Bangera” in Pakistan.[4,5] This species is used as antidiabetic, sedative, digestive aid, and antianemic in Turkish folk medicine[6,7] Antidiabetic, antihepatotoxic, antinociceptive, antiinflammatory, and antiulcerogenic activities of *G. olivieri* were proved.[4,8–10] In the Uzbekistan Republic, it has been used to cure diarrhea, common cold, stomachache, and indigestion.[11] Antibacterial, antifungal, antihypertensive, toxicological, and diuretic effects of the plant were studied in Pakistan.[12–14] Although there are many other studies on *G. olivieri* about finding new compounds by chemical methods[15–17] and tissue culture applications,[18] there is not any data on anatomical features of *G. olivieri*.

Metcalfe and Chalk[19] reported on general anatomical studies on Gentianaceae based on Perrot[20] and Martens.[21] Wood[22,23] and seed structure,[24] pollen morphology and ultrastructural diversity[25] were investigated. Root structure was investigated by Budimir *et al*.[26] and Sottnikova and Lux[27] on *G. lutea* and *G. asclepiadea*, respectively. In this study, the anatomical characteristics of *Gentiana olivieri* Griseb, were studied to provide oppurtunities for further studies.

## MATERIAL AND METHODS

**Figure 1:**
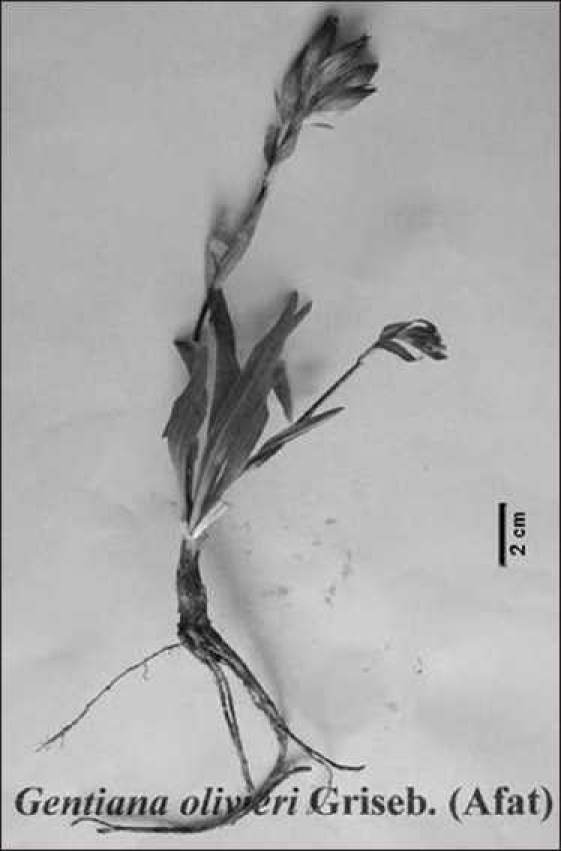
*Gentiana olivieri* Griseb

Flowered plants were collected in summer from Sivas (Turkey) [Figure 1]. Identification of the specimen was determined by the key presented in “Flora of Turkey and the East Aegean Islands”.[1] Voucher specimen were deposited in the Herbarium of the Faculty of Science, Ankara University, Turkey (Acronym: ANK).

Collected living materials were fixed in 70% ethyl alcohol for anatomical studies. The paraffin wax method was applied for preparing cross sections of mature roots, stems, and leaves. Cross sections, 15–20 µm were made with a Leitz sliding microtome[28,29] and by hand. Free-hand sections were taken from leaves surface. Safranin and Safranin-Fast Green, Safranin-Crystal Violet double stains were used for a better understanding of some anatomical structures of the sections.[30]

Sections were examined with Leica DM LS2 light microscope and photographed by Leica DFC 320 digital camera using Image Manager 50 program. Various measurements were performed on microscopic images.

## Results

### Root

**Figure 2:**
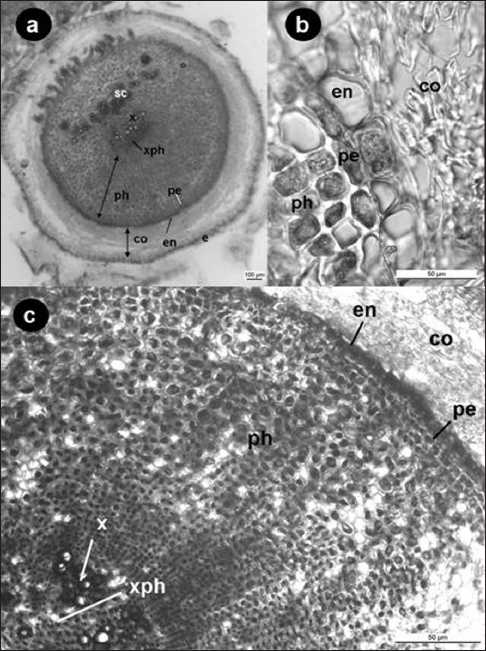
a, b. Cross-section of root c. Central cylinder of the root. Epidermis (e), cortex (co), sclerenchyma (sc), endodermis (en), pericycle (pe), phloem (ph), xylem (x), intraxylary phloem (xph).

The root system of *G. olivieri* consisted of thick, fleshy storage roots from which fibrous adventitious roots grew out, like *G. asclepiadea*.[27] Root hairs were absent. The outer surface of root was covered by a single layer epidermis, which consisted of thin layered, crushed, and broke up isodiametric shaped cells. Epidermis was papillose. Cortex was multi-layered. Its cells were also crushed and had large intercellular spaces. Mycorrhiza was present in the root system.[19] There were sclerenchyma cells and bundles in the area of the contact point of adventitious roots [Figure 2a]. Endodermis was well defined and consisted of longitudinally elongated cells. Casparian strips were observed in anticlinal walls of endodermis cells [Figure 2b]. Some of the cells of endodermis were completely thickened. Pericycle was 1–2 layered. The central cylinder was composed of multi-layered cells, which had radial symmetry. Vascular bundles were collateral, diarch, and had not a cambium. Very broad parenchymatic phloem was present in interfascicular area. These cells also showed radial symmetry [Figure 2a,c]. Sieve-tube elements and companion cells were very rare and adjacent to xylem zone. Intraxylary phloem between xylems was present in pith as mentioned in the “Anatomy of the Dicotyledones”.[19]

### Stem

**Figure 3:**
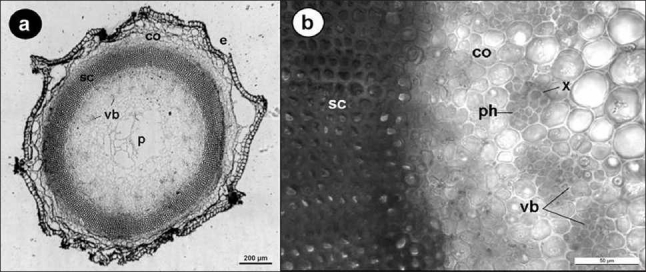
a, b. Cross-section of stem. Epidermis (E), cortex (C), sclerenchymatic ring (ScR), vascular bundles (Vb), pith (P), xylem (X), phloem (Ph)

There was a cuticular layer, and 6.05 ± 1.7 µm thick. A single layer epidermis cells were isodiametric. Epidermis provided with 4–5 wings with the participation of parenchymatic cells of the cortex tissue [Figure 3a]. Cortex cells, which had large intercellular spaces were 3–6 layered and 4.29 ± 1.7 × 6.87 ± 2.34 µm in size. Underneath the epidermis, there was sclerenchymatic ring consisting of small, multi-layered cells surrounding the central cylinder, which was composed of parenchymatic tissue with thin primary cell walls (Thickness of sclerenchymatic ring was 205.6 ± 43.26 µm). Vascular bundles were in many (41–46) small groups [Figure 3b]. The xylem comprised tracheid with a regular single strand. The phloem was 1–2 layered and consisted of sieve-tube elements. Interfascicular cambium was absent and arrengement of vascular bundles were collateral. Bundles were surrounded by bundle sheath. There were large parenchymatic cells in pith. Crystalline structures were not observed.

### Leaf

**Figure 4:**
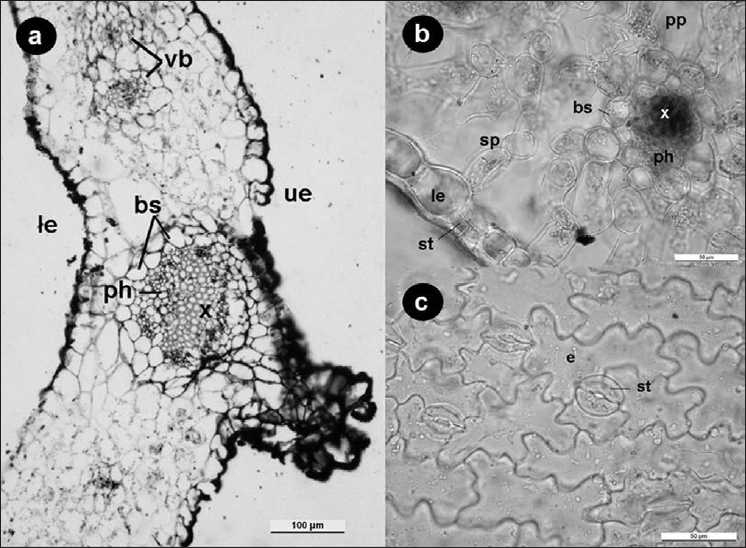
a, b. Cross-section of leaf c. Surface section of leaf. Upper epidermis (ue), xylem (x), phloem (ph), vasculary bundle sheath (bs), vascular bundles (vb), lower epidermis (le). Stoma (st), spongy parenchyma (sp), palisade parenchyma (pp), epidermis (e)

In a cross section of the leaf, cuticle of the lower epidermis was thicker than of the upper one [Table 1]. Both epidermis cells were almost isodiametric, rectangular, and papillose [Figure 4a]. Although mesophyll consisted of nearly same type cells, palisade, and spongy parenchyma cells were differenciated. Palisade tissue seemed similar to spongy parenchyma cells because palisade tissue spreaded out infrequently in the leaf and had relatively large intercellular spaces. Cells under the upper epidermis, which were perpendicular to the surface, thin, and elongated longitudinally pointed out that these were palisade parenchyma cells [Figure 4b]. Palisade parenchyma cells were 1–3 layered whereas spongy parenchyma cells were 4–5 layered. Vascular bundles except the midrib were embedded in mesophyll. They had collateral structure type. There was not sclerenchymatic tissue in the bundles but parenchymatic bundle sheath was present [Figure 4ab].

**Table 1:** Measurements of cells and structures of various tissue of *G. olivieri* (SD, standard deviation

	Length (µm)	Width (µm)
Root		
Epidermal cells	19.91 ± 3.91	51.27 ± 15.23
Cortex cells	12.32 ± 1.61	28.38 ± 5.23
Endodermis cells	23.03 ± 9.20	37.83 ± 10.09
Pericycle cells	10.23 ± 2.40	31.47 ± 7.35
Stem		
Cuticle	6.05 ± 1.70	
Epidermal cells	14.81 ± 9.59	17.18 ± 11.16
Screlenchymatic ring		205.6 ± 43.26
Leaf		
Cuticle- upper epidermis	9.81 ± 1.35	
Upper epidermal cells	31.62 ± 3.62	29.34 ± 1.70
Cuticle- lower epidermis	13.23 ± 2.06	
Lower epidermal cells	28.37 ± 4.19	31.34 ± 5.62
Guard cells	7.19 ± 1.43	32.88 ± 2.46

In a surface section, epidermis cells were elongated longitudinally and in the long sides of the cells had large waves (sinuate). Stomata were anomocytic (Ranunculaceous) type and guard cells were surrounded by 3–4 subsidiary cells [Figure 4c]. Some stomata showed similar features with anysocytic (Cruciferous) type. The upper epidermis had less stomata (Amphistomatic). Stomata were located on the same level as epidermis cells.

## DISCUSSION

Anatomical features of *Gentiana olivieri* have not been investigated until now. Studies on the anatomy of Gentianaceae have limited, although recent investigations have been conducted on the anatomical structure of the wood. Jansen and Smets[22] investigated wood anatomy of *Anthocleista, Fagraea, Lisianthius, Macrocarpaea, Nuxia, Symbolanthus* and *Tachiadenus*, which belonged to Gentianales. In the radial and cross sections, existence of vestured pits in aperture or cavity of tracheids were proved. Carlquist and Grant[23] investigated stem wood anatomy and displayed the differences of *Symbolanthus macranthus, Tachia occidentalis*, and 17 species of *Macrocarpaea*, which were included Helieae tribus.

Comparison of the anatomic features of *G. olivieri* was made according to the “Anatomy of the Dicotyledones”[19], because anatomical investigations limited on Gentianaceae. Anatomical characters of species were observed to have been similar to usual features of Gentianaceae family. It was determined that the most important anatomical difference between Gentianoideae and Menyanthoideae sub-families was existence of intraxylary phloem in the roots of Gentianoideae. This characteristic was observed on *G. olivieri* which was including Gentianoideae. Adventititous roots and rays were absent as mentioned by family features. Vessels were present as a single or groups. Endodermis was a single layered, elongated longitudinally and had Casparian strips on radial walls in *G. olivieri* whereas some *Swertia* species had 2 layered endodermis.

Papillose epidermis on stem was usual anatomical feature of Gentianoideae. In stems of *G. olivieri*, there were two rings consisted of sclerenchymatic outside and many vascular bundles inside, like *G. asclepiadea* and *G. septemfida*. Whereas the pith of *G. pneumonanthe* was partially sclerotic, the piths of *Lehmanniella* and *Senea* were completely sclerotic but the pith was composed of parenchymatic cells in *G. olivieri*. Though crystals were found in cortex and in pith of *Enicostemma*, it was not found in *G. olivieri*.

## CONCLUSION

Our findings suggested that the leaf anatomy of *G. olivieri* exhibited a papillose structure on the epidermis and anomocytic type stomata but none of the sclerenchymatic cells in vascular bundles, as also observed in Gentianaceae.
